# Reproducibility of regional- and global left-ventricular strain analyzes using tagging and feature tracking techniques - a cardiac magnetic resonance imaging study in healthy volunteers

**DOI:** 10.1186/1532-429X-18-S1-P58

**Published:** 2016-01-27

**Authors:** Dominik Buckert, Nils Dyckmanns, Wolfgang Rottbauer, Peter Bernhardt

**Affiliations:** Innere Medizin II, Uniklinik Ulm, Ulm, Germany

## Background

Techniques for the evaluation of regional- and global left-ventricular wall motion parameters based on strain analyzes are emerging in cardiac magnetic resonance (CMR) imaging. Some of these techniques, such as tagging, require dedicated CMR sequences at the time of the examination. Other approaches are based on post-processing of routine imaging studies such as feature-tracking. Common to all is the need for specialized software solutions. Objective of our study was the evaluation of a tagging and a feature-tracking approach with regard to reproducibility and robustness. We therefore examined a cohort of healthy volunteers under rest conditions and during pharmacological exercise.

## Methods

Our study cohort consisted of 40 healthy volunteers aged between 19 and 31 years (mean age: 24 ± 3.07 years), 23 of them were women (57.5%). CMR imaging was conducted on a 1.5 Tesla whole-body scanner (Intera, Philips Medical Systems, Best, the Netherlands) using a 32-channel phased-array surface receiver coil. To simulate physical exercise, a pharmacological stress protocol based on dobutamine was applied. Standard cine images were assessed using a balanced steady-state free precession sequence. Tagging images were generated using a spatial modulation of magnetization sequence. Cine images were analyzed by the feature tracking module of cvi42, Circle Cardiovascular Imaging, Calgary, Canada. For the evaluation of tagging images, the software solution HARP, Diagnosoft, Durham, USA, was applied. Analyzes were performed offline by two experienced readers who were blinded to the results of each other.

## Results

Tagging-derived parameters with highest reproducibility (intraclass correlation, IC; inter-rater agreement, IRA) were mean circumferential peak strain (rest: IC: .92 [.81; .96]; IRA: .82 [.64; 1.00]; mid stress: IC: .89 [.78; .95]; IRA: .81 [.70; .92]; max stress: IC: .94 [.87; 97]; IRA: .85 [.76; .94]) and mean time to circumferential peak strain (rest: IC: .74 [.50; .86]; IRA: .79 [.62; .95]; mid stress: IC: .93 [.86; .96]; IRA: .78 [.67; .88]; max stress: IC: .67 [.37; 83]; IRA: .63 [.43; .84]). Other parameters showed in part unacceptable low reproducibility, especially during exercise. Feature-tracking-derived parameters in general showed higher reproducibility. Most robust parameters were again mean circumferential peak strain (rest: IC: .96 [.71; .99]; IRA: .93 [.90; .97]; mid stress: IC: .95 [.78; .98]; IRA: .83 [.73; .93]; max stress: IC: .82 [.47; .93]; IRA: .72 [.59; .86]) and mean time to circumferential peak strain (rest: IC: .92 [.85; .96]; IRA: .87 [.78; .96]; mid stress: IC: .95 [.91; .97]; IRA: .86 [.78; .94]; max stress: IC: .89 [.79; 94]; IRA: .76 [.62; .90]).

## Conclusions

We found mean circumferential peak strain and mean time to circumferential peak strain the parameters with highest reproducibility, independent whether derived from tagging or feature-tracking. In general, feature-tracking delivered more robust values, especially under exercise conditions.

